# Target Product Profile for a Machine Learning–Automated Retinal Imaging Analysis Software for Use in English Diabetic Eye Screening: Protocol for a Mixed Methods Study

**DOI:** 10.2196/50568

**Published:** 2024-03-27

**Authors:** Trystan Macdonald, Jacqueline Dinnes, Gregory Maniatopoulos, Sian Taylor-Phillips, Bethany Shinkins, Jeffry Hogg, John Kevin Dunbar, Ameenat Lola Solebo, Hannah Sutton, John Attwood, Michael Pogose, Rosalind Given-Wilson, Felix Greaves, Carl Macrae, Russell Pearson, Daniel Bamford, Adnan Tufail, Xiaoxuan Liu, Alastair K Denniston

**Affiliations:** 1 Ophthalmology Department Queen Elizabeth Hospital Birmingham University Hospitals Birmingham National Health Service Foundation Trust Birmingham United Kingdom; 2 Academic Unit of Ophthalmology, Institute of Inflammation and Aging College of Medical and Dental Sciences University of Birmingham Birmingham United Kingdom; 3 National Institute for Health and Care Research Birmingham Biomedical Research Centre Birmingham United Kingdom; 4 School of Business University of Leicester Leicester United Kingdom; 5 Warwick Medical School University of Warwick Coventry United Kingdom; 6 Population Health Sciences Institute Faculty of Medical Sciences The University of Newcastle upon Tyne Newcastle United Kingdom; 7 NHS England Leeds United Kingdom; 8 Population Policy and Practice University College London Great Ormond Street Institute of Child Health London United Kingdom; 9 Moorfields Eye Hospital NHS Foundation Trust London United Kingdom; 10 Lay member Oxford United Kingdom; 11 Alder Hey Children’s Hospital Alder Hey Children’s Hospital NHS Foundation Trust Liverpool United Kingdom; 12 Hardian Health London United Kingdom; 13 St. George’s University Hospitals National Health Service Foundation Trust London United Kingdom; 14 National Institute for Health and Care Excellence London United Kingdom; 15 Faculty of Medicine School of Public Health Imperial College London London United Kingdom; 16 Nottingham University Business School University of Nottingham Nottingham United Kingdom; 17 Medicines and Healthcare Products Regulatory Agency London United Kingdom; 18 Institute of Ophthalmology University College London London United Kingdom; 19 Centre for Regulatory Science and Innovation Birmingham Health Partners Birmingham United Kingdom; 20 National Institute for Health and Care Research Biomedical Research Centre at Moorfields and University College London Institute of Ophthalmology London United Kingdom

**Keywords:** artificial intelligence, design, developers, diabetes mellitus, diabetic eye screening, diabetic retinopathy, diabetic, DM, England, eye screening, imaging analysis software, implementation, machine learning, retinal imaging, study protocol, target product profile

## Abstract

**Background:**

Diabetic eye screening (DES) represents a significant opportunity for the application of machine learning (ML) technologies, which may improve clinical and service outcomes. However, successful integration of ML into DES requires careful product development, evaluation, and implementation. Target product profiles (TPPs) summarize the requirements necessary for successful implementation so these can guide product development and evaluation.

**Objective:**

This study aims to produce a TPP for an ML-automated retinal imaging analysis software (ML-ARIAS) system for use in DES in England.

**Methods:**

This work will consist of 3 phases. Phase 1 will establish the characteristics to be addressed in the TPP. A list of candidate characteristics will be generated from the following sources: an overview of systematic reviews of diagnostic test TPPs; a systematic review of digital health TPPs; and the National Institute for Health and Care Excellence’s Evidence Standards Framework for Digital Health Technologies. The list of characteristics will be refined and validated by a study advisory group (SAG) made up of representatives from key stakeholders in DES. This includes people with diabetes; health care professionals; health care managers and leaders; and regulators and policy makers. In phase 2, specifications for these characteristics will be drafted following a series of semistructured interviews with participants from these stakeholder groups. Data collected from these interviews will be analyzed using the shortlist of characteristics as a framework, after which specifications will be drafted to create a draft TPP. Following approval by the SAG, in phase 3, the draft will enter an internet-based Delphi consensus study with participants sought from the groups previously identified, as well as ML-ARIAS developers, to ensure feasibility. Participants will be invited to score characteristic and specification pairs on a scale from “definitely exclude” to “definitely include,” and suggest edits. The document will be iterated between rounds based on participants’ feedback. Feedback on the draft document will be sought from a group of ML-ARIAS developers before its final contents are agreed upon in an in-person consensus meeting. At this meeting, representatives from the stakeholder groups previously identified (minus ML-ARIAS developers, to avoid bias) will be presented with the Delphi results and feedback of the user group and asked to agree on the final contents by vote.

**Results:**

Phase 1 was completed in November 2023. Phase 2 is underway and expected to finish in March 2024. Phase 3 is expected to be complete in July 2024.

**Conclusions:**

The multistakeholder development of a TPP for an ML-ARIAS for use in DES in England will help developers produce tools that serve the needs of patients, health care providers, and their staff. The TPP development process will also provide methods and a template to produce similar documents in other disease areas.

**International Registered Report Identifier (IRRID):**

DERR1-10.2196/50568

## Introduction

### Diabetic Eye Screening and Its Delivery in the United Kingdom

Diabetic eye screening (DES) aims to prevent vision loss from diabetic retinopathy (DR), a sight-threatening microvascular complication of diabetes [1]. In the United Kingdom, people with diabetes aged 12 years or older undergo yearly fundus photography with images interpreted by human graders to detect and assess the severity of DR. This allows detection of DR in its early asymptomatic stages, facilitating early referral to hospital eye services where treatment is more effective [2,3]. DES is associated with improved clinical [4] and economic [5] outcomes and a reduction in blindness from DR since its introduction in 2003 [6]. However, DES is both labor and resource intensive [7], and costs are expected to increase with a projected rise in diabetes prevalence [8]. In the United Kingdom, grading is undertaken by experienced professionals in a quality-assured and controlled multilevel system [9] with a high sensitivity for sight-threatening diseases [10]. However, grading represents a significant cost despite the fact that the majority of people with diabetes have no evidence of DR or only mild disease [11] and are at low risk of visual loss in the near term [12].

### Automated Retinal Imaging Analysis Software

DES grading is considered a promising use case for automation, and indeed, DES in Scotland has used automated retinal imaging analysis software (ARIAS) since 2012 [13,14]. This software uses “symbolic” artificial intelligence (AI) with grading “rules” programmed by humans [15] and is used in a primary grader role. The Scottish “autograder” has demonstrated a sensitivity of 90.5% (95% CI 89.3%-91.6%) and specificity of 67.4% (95% CI 66%-68.8%) for referable disease or ungradable images [13], with 50% of images it graded in 2018 requiring no further human review [16]. It is, however, unable to process the optic disc–centered images acquired in screening protocols elsewhere in the United Kingdom and has not been adopted outside Scotland [17].

Subsequent advances in AI, most notably in machine learning (ML), where AI “learns” patterns from data it is presented as opposed to being programmed by humans, have given rise to a newer generation of machine learning–automated retinal imaging analysis software (ML-ARIAS). These have demonstrated better performance than symbolic tools on routinely collected UK screening images, including disc-centered photos. Heydon et al [18] found ML-ARIAS was able to achieve sensitivity and specificity of 95.7% (95% CI 94.8%-96.5%) and 54% (95% CI 53.4%-54.5%), respectively, for referable disease or ungradable images in a large sample taken from routine screening in 3 UK programs. A health technology assessment has also estimated that ML-ARIAS in a triage or primary grader role could be cost-effective and cost-saving compared to purely manual grading [17]. Consequently, there have been calls for ML-ARIAS’s adoption into DES in the United Kingdom [19].

### Bridging the Implementation Gap

In response to these calls, the UK National Screening Committee (NSC), an independent scientific advisory group that advises UK governments on screening programs, commissioned a review to ascertain whether ARIAS should be more widely adopted in 2021 [19]. While the review found evidence suggesting ML-ARIAS to be safe, accurate, and cost-effective, it also highlighted evidence gaps that prevented a recommendation for their use. The review highlighted that the acceptability of ML-ARIAS with patients and health care professionals was unknown, as was their real-world performance post implementation, recognizing that this may be affected by a range of clinical and technical factors. While research to address these evidence gaps is currently ongoing [20], the review’s conclusions reflect an awareness in the wider field of health care AI that evidence of good diagnostic accuracy cannot predict the impact tools will have post deployment. This awareness contributes toward a growing “implementation gap” [21,22] or “chasm” [23] in health care AI, with increasing numbers of tools failing to be adopted into clinical use following promising research. This situation has a number of negative consequences, such as significant opportunity cost, with tools’ development requiring significant public [24] or private investment [25], as well as a failure for patients, health care professionals, or systems to realize the benefits tools may offer for a lack of appropriate evidence generation.

A similar situation has existed in the pharmaceutical and medical device industries for decades. Despite significant investment in research and development, few biomarkers, drugs, or devices are ever adopted into routine clinical care, often failing to achieve regulatory approval or demonstrate sufficient clinical or economic benefits to health care providers [26,27]. To combat this situation, pharmaceutical companies began to outline the characteristics necessary for a successful product in the earliest stages of development, summarizing these in “target product profiles” (TPPs). These documents often incorporate considerations essential to successful clinical use but not covered in traditional research, such as interaction with existing care pathways and resource constraints. TPPs are used to guide research development as part of a broader “quality by design” approach that has been encouraged by regulatory agencies [28,29] and widely adopted in industry with notable successes [30,31]. TPPs have since been used by governments [32], health care providers [33], and nonprofit organizations [34] for drugs [35,36] and medical devices [37], particularly in the context of infectious diseases [38,39] and the developing world [40,41].

### Aim

This study aims to produce a TPP for an ML-ARIAS for use in English DES (E-DES), enabling the development of ML tools that match the needs of people with diabetes, health care professionals, regulators, and providers. This work will focus on the English context, as variations in the commissioning and organization of DES between UK nations may affect the stakeholders needed as well as the TPP’s final contents. However, we hope this work will be valuable for screening programs in other nations in the United Kingdom and beyond.

### Objectives

The objectives of this study are to (1) identify which characteristics should be addressed in a TPP for an ML-ARIAS (this will be achieved through an overview of systematic reviews of TPPs, a systematic review of digital health TPPs, and by extracting standard’s from the National Institute of Health and Care Excellence’s (NICE) Evidence Standards Framework for Digital Health Technologies (ESF); (2) draft specifications for an ML-ARIAS TPP through semistructured interviews with members of key stakeholder groups; and (3) validate the contents of a TPP for an ML-ARIAS through a modified Delphi consensus study.

## Methods

### Ethical Considerations

Ethical approval for this study has been granted by the University of Birmingham Institutional Research Board (ERN_2023-0620). The study will be undertaken in accordance with the Helsinki Declaration of 1975, as revised in 2000. Informed consent will be sought from participants. The interviews and consensus meeting will be recorded and transcribed, with participants given the opportunity to opt out with deletion of their responses up to a week after their interview or meeting date, after which their responses will be anonymized and therefore no longer attributable to them. Recordings of the interview and consensus meeting will be deleted following transcription, with the anonymized transcripts stored on secure institutional research servers for up to 10 years. Delphi participants will be informed that their responses are anonymized and therefore cannot be deleted following submission. Contact details they provide in order to be able to participate in the consensus meeting will be stored on secure servers until the end of the study, after which they will be deleted.

People with diabetes participating in the study advisory group (SAG) and consensus meeting will have their travel costs reimbursed as well as their time reviewing documents or attending meetings at £25 (US $31.57) per hour in line with National Institute for Health and Care Research (NIHR) guidance [42,43].

### Identifying Key Stakeholder Groups

The successful use of ML-ARIAS in E-DES will require the approval, collaboration, and consent of a diverse range of stakeholder groups. To ensure our TPP is both comprehensive and authoritative it is therefore important to incorporate the knowledge and opinions of all these groups in its development.

A recent qualitative systematic review on stakeholder perspectives of clinical AI implementation identified the following key stakeholder groups influencing implementation: patients, carers, and the public; health care professionals; health care managers and leaders; regulators and policy makers; and developers [44]. Mapped to our use case and context, these broad stakeholder groups can be further divided as follows: people with diabetes, carers, and the public; E-DES technicians and graders, as well as ophthalmologists (health care professionals); managers of E-DES services provided by either the NHS or independent providers (health care managers and leaders); the Medicines and Healthcare Products Regulatory Agency (MHRA), NICE, the Care Quality Commission (CQC), and NSC (regulators and policy makers); and ML-ARIAS developers. Our study will aim to encompass the opinions of all these groups as outlined below.

### Study Delivery and Oversight

#### Study Management Group

A study management group (SMG) led by the chief investigator AKD will be responsible for the design and delivery of the study.

##### Recruitment

SMG members will be recruited from the host institution to facilitate ease of communication and regular meetings.

##### Sampling

The SMG will include experts in the relevant clinical, scientific, and methodological domains, project management support, and a person with diabetes.

#### Study Advisory Group

The SMG will be supported by a SAG, which will bring additional breadth of expertise from a range of key stakeholder groups. The SAG will provide critical feedback and advice throughout the project in quarterly project review meetings and at specific junctures outlined in the protocol and ad hoc.

##### Recruitment

SAG recruitment strategies will be tailored to individual stakeholder groups. People with diabetes will be recruited through diabetes charities. Clinical and methodological experts with previous experience developing TPPs will be identified through their publications in the field. Representatives of relevant regulatory and policy bodies will be recruited through their organizations (NSC, MHRA, NICE, and CQC).

##### Sampling

The SAG will aim to recruit at least 2 representatives from each of the broad stakeholder groups previously identified, with the exception of ML-ARIAS developers, omitted to avoid bias arising from conflict of interest. Experts in TPP development and health economics will also be invited to the SAG to add a greater breadth of expertise.

#### User Group

A TPP user group (UG) consisting of ML-ARIAS developers will be assembled to provide feedback on the TPP as outlined below to ensure feasibility is factored into the development process.

##### Sampling

The UG will consist of only ML-ARIAS developers.

##### Recruitment

Developers with ML-ARIAS approved by British, European, Australian, or US medical device regulators will be invited to participate, along with those with technologies included in the UK NSC’s 2021 review.

### Phase 1: Literature Reviews to Establish the Scope of an ML-ARIAS TPP

**Figure 1:**
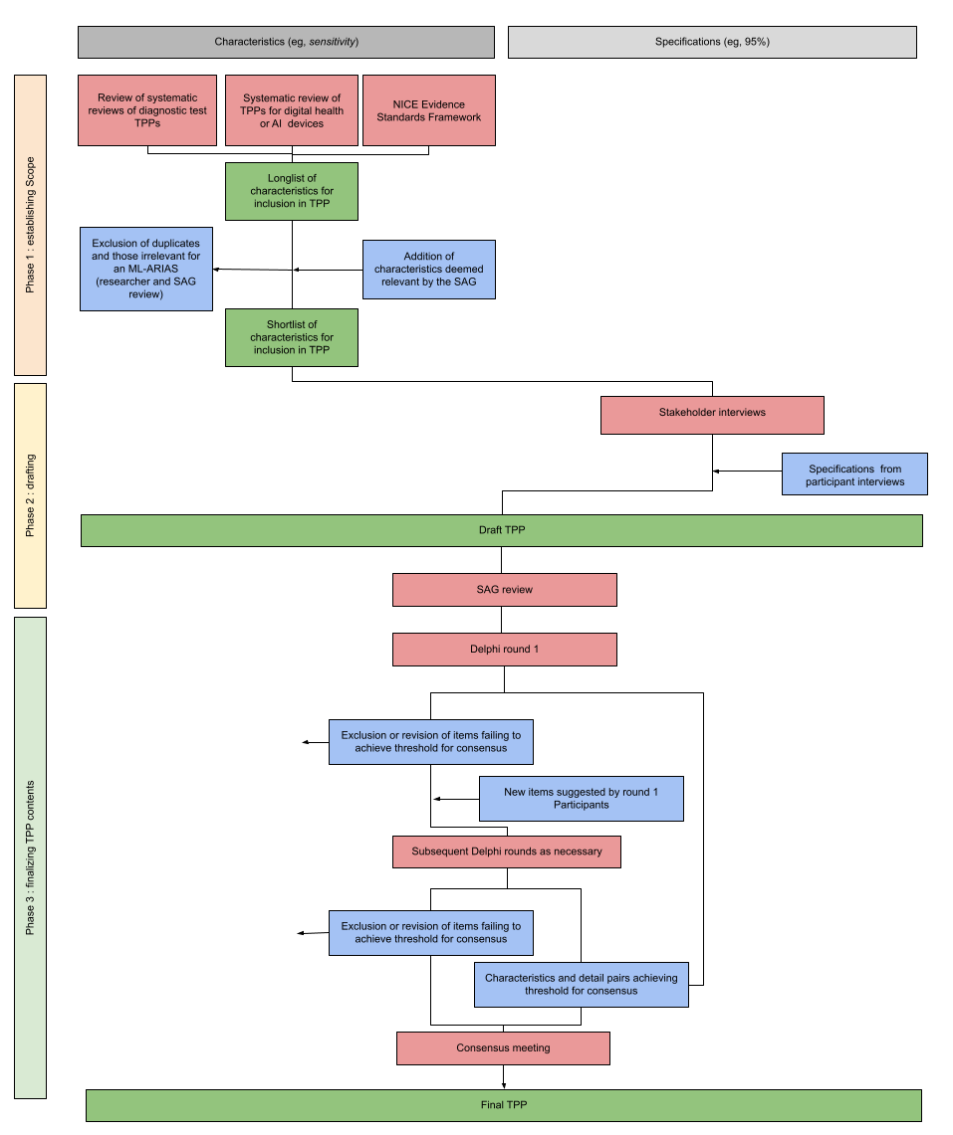
Diagram of the machine learning–automated retinal imaging analysis software (ML-ARIAS) target product profile (TPP) development process. AI: artificial intelligence; NICE: National Institute for Health and Care Excellence; SAG: study advisory group.

See Figure 1 for an overview of the ML-ARIAS TPP development process.

#### Rationale

This phase will seek to establish what characteristics a TPP for an ML-ARIAS should address in order to be both relevant and comprehensive. A long list of potential characteristics for inclusion in an ML-ARIAS TPP will be generated through 2 reviews. These will be built on the work of Cocco et al [45], who published a list of all characteristics previously reported in diagnostic test TPPs in a 2020 systematic review.

#### Overview of Systematic Reviews of Target Product Profiles for Diagnostic Tests

This will seek to identify all systematic reviews of diagnostic test TPPs with the aim of extracting the characteristics reported in each review or the TPPs they capture. Based on a scoping search, we anticipate this review will include the systematic review by Cocco et al [45] and any new resources published since.

#### Systematic Review of TPPs for Digital Health Tools

As Cocco et al’s [45] review did not include any TPPs for digital health tools, our long list of potential characteristics may therefore omit characteristics unique to these tests. The purpose of this review is to identify all TPPs published for digital health tools (including AI) and extract the characteristics they report. This will include TPPs for therapeutic as well as diagnostic devices.

Multimedia Appendix 1 includes MEDLINE search strategies for both reviews, developed in consultation with an information specialist and using validated search filters where possible [45-47]. Strategies for other bibliographic databases will be adapted from these, as will a web search strategy using a systematic method previously reported [48]. The 2 reviewers (TM and JH) will perform a title and abstract screening, then a full-text review to assess eligibility. Disagreements will be arbitrated by the senior investigator (AKD).

#### Generating AI-Specific Characteristics from NICE’s Evidence Standards Framework for Digital Health Technologies

##### Rationale

As scoping searches for digital health TPPs have returned very few TPPs for AI and specifically ML tools, relying on the above reviews alone to determine the scope of our TPP may risk omitting characteristics unique to these devices and essential for their successful deployment. To generate AI-specific characteristics, we will therefore adapt standards from NICE’s ESF for digital health technologies, a gold standard tool designed to evaluate digital health technologies for UK commissioning decisions. This was revised in 2022 to encompass AI devices, with the new AI standards being generated through a multistakeholder Delphi consensus process [49,50]. The 2 reviewers (TM and JH) will review the ESF, extracting relevant standards, with disagreements resolved by discussion or arbitration by the senior investigator (AKD). The extracted standards will be adapted into characteristics and added to the long list from the earlier reviews.

##### Shortlisting Characteristics for Inclusion in a Draft TPP

The list of candidate characteristics will be reviewed by the SMG to remove any irrelevant to the use case or proposed technology and consolidate the remainder, removing any duplicates. Disagreements between SMG members will be resolved through discussion and arbitration by the senior investigator (AKD) if necessary.

The remaining characteristics will then be mapped to the nonadoption, abandonment, scale-up, spread, and sustainability (NASSS) framework [51], a tool outlining the interacting complexity of factors at the policy, organizational, and practice level that may influence the successful implementation of digital health technology [52]. The NASSS will help group the characteristics into broader themes and help identify factors affecting successful implementation that they do not cover.

The included characteristics will then be presented to the SAG, along with those excluded and the NASSS domains not covered by the shortlist. SAG members will be asked to identify any characteristics incorrectly excluded and approve a final shortlist of characteristics for subsequent research phases through discussion or vote.

### Phase 2: Drafting the TPP

#### Scoping Interviews

A TPP typically includes a table of characteristics and their specifications. Characteristics might include “target population” with “people with diabetes aged 12 years or older” as its associated specification. The drafting of specifications for each of the characteristics shortlisted in phase 1 will be informed by semistructured interviews with participants from each key stakeholder group previously identified. The final output of phase 2 will be a full draft TPP for entry into a modified Delphi consensus study in phase 3.

#### Sampling

Purposive sampling will initially be used with the aim of interviewing at least 1 individual from all the stakeholder groups relevant to the use case previously identified, with the exception of ML-ARIAS developers. As the interviews progress, theoretical sampling will be used to better explore areas of disagreement or characteristics that have not been addressed in previous interviews. Snowball sampling will also be used, with participants invited to suggest potential interviewees. An approximate target of 20 participants has been set; however, the scale of recruitment will be refined by the SMG as the interviews progress.

#### Recruitment

People with diabetes will be recruited through diabetes charities and offered compensation for their time in accordance with NIHR guidelines [42,43]. Clinical and methodological experts, as well as industry representatives with previous knowledge or experience of AI in DES or TPPs, will be identified through their publications in the field. Representatives of relevant regulatory and policy bodies will be recruited by contacting their organizations (NSC, MHRA, and NICE). It is anticipated that members of the SAG will facilitate the recruitment of key stakeholders in their respective stakeholder groups.

#### Data Collection

Ahead of the interviews, participants will be sent information in plain English on E-DES, AI, and TPPs.

The interviews will be conducted over video teleconferencing software (Zoom; Zoom Video Communications, Inc). Interview participants will initially be asked to provide demographic data, including stakeholder groups, location, age, sex, gender, and ethnicity. A preprepared topic guide informed by the shortlist of characteristics produced in phase 1 will be developed and used to encourage a free-flowing conversation between interviewer and interviewee focused on the latter’s opinions, priorities, and concerns regarding the adoption of ML-ARIAS in E-DES. The topic guide will be iterated as the interviews progress to ensure all implementation considerations are covered and adapted to different stakeholder groups to facilitate meaningful participation.

All the interviews will be conducted by 2 clinicians (TM and JH) with previous training and experience in qualitative interviewing. They will present themselves as researchers during the interviews so as not to introduce bias to the data collected [53]; however, as their status may become apparent during the interview and can be discovered on the internet, they will use reflective journaling to identify and mitigate against any effects this may have on data collection [54]. Both interviewers will take field notes during and after the interviews to provide context for the data analysis.

#### Data Analysis

All the interviews will be digitally recorded and transcribed verbatim. Data analysis will begin after the first interview and proceed in parallel with the remainder. One researcher (TM) will code the interview transcripts using qualitative analysis software (NVivo, Lumivero). The coded data will then be mapped to the shortlist of characteristics produced from phase 1, using this as a framework to aid analysis [55].

The data collected will be reviewed throughout the collection period by the SMG to identify and manage biases in sampling, data collection, analysis, and interpretation. The data will be used to iterate the topic guide, recruitment, and sampling strategies to allow the exploration of problematic issues, divergent views between or within stakeholder groups, and implementation considerations not previously explored.

On completion of the data analysis, the SMG will draft characteristics’ specifications based on the analyzed data. An initial draft TPP will be presented to the SAG for review and approval before entry into phase 3.

### Phase 3: Finalizing the TPP

#### Internet-Based Delphi Consensus Rounds

The draft TPP will be entered into an internet-based Delphi study using purpose-made software (Qualtrics XM; Qualtrics). The contents and form of the Delphi survey will be coproduced by the SMG and SAG with the close involvement of both groups’ patient representatives. It will also be piloted to ensure it is broadly comprehensible and accessible.

At the start of each Delphi round, participants will be presented with information in plain English on E-DES, AI, and TPPs. In subsequent rounds after round 1, participants will also be provided with a report of the previous round (see Data Analysis section below). Each round will remain open for a minimum of 2-4 weeks, and it is expected that at least 2 rounds will be conducted. Extensions to rounds’ time frames and further rounds may be considered in consultation with the SAG.

#### Sampling

In line with similar studies successfully conducted in other contexts [56-58], we aim to recruit more than 100 respondents overall, with at least 10 from each broader stakeholder group previously outlined, including ML-ARIAS developers. Completed returns will be monitored by the stakeholder group, and further measures taken targeted at individual groups to boost recruitment if necessary. These may include targeted email or newsletter reminders.

#### Recruitment

People with diabetes will be recruited by asking diabetes charities to disseminate a link to the Delphi survey among their members. Similarly, the British Association of Retinal Screeners will be asked to disseminate a link among their members to recruit DES professionals. The SMG and SAG will be asked to disseminate a survey link to their wider networks to recruit regulators, clinicians, and academics. ARIAS developers with technologies previously identified by the UK NSC’s Rapid Review will be invited to participate, as will those receiving funding from the NHS AI Health and Care Award.

#### Data Collection

Participants will be asked which stakeholder groups they belong to, their geographic location, age, sex, gender, and ethnicity. In each round, participants will be asked to score each characteristic and specification on a Likert scale ranging from 1 to 5, with 1 being “definitely exclude” and 5 being “definitely include.” Recognizing that some stakeholder groups may not have the expertise to confidently score characteristic and specification pairs in some areas, participants will be given the option to omit pairs or identify other stakeholder groups they would be comfortable making this decision on their behalf. Free-text boxes will be provided to comment on characteristic and specification pairs or suggest edits. Another box at the end of the survey will provide an opportunity for participants to suggest entirely new characteristic and specification pairs.

#### Data Analysis

After each round, the responses will be aggregated and analyzed. A report will be produced summarizing response rates and characteristic and specification pairs scores descriptively, with scores broken down by subgroup. Free-text responses will be imported into qualitative analysis software and coded.

Consensus will be defined as >70% of respondents scoring a characteristic and specification pair 4-5 and <15% 1 in any stakeholder group. Characteristic and specification pairs exceeding this threshold will exit the survey process and proceed to the consensus meeting for discussion. Those not meeting this threshold will be revised by the SMG, taking into account their scores in previous rounds by different stakeholder groups and their coded free-text responses. The revised characteristic and specification pairs will then enter subsequent rounds, along with any new items suggested by participants in the previous round.

### Consensus Meeting

The Delphi results will be reviewed, and the final TPP contents will be agreed upon at an in-person consensus meeting.

Before the consensus meeting, feedback will be sought from members of the UG. This feedback, along with the Delphi round results broken down by stakeholder group, will be sent to consensus meeting participants ahead of the meeting.

The consensus meeting itself will be led by an experienced facilitator. At the meeting, participants will be presented with each characteristic and specification pair in turn, and an opportunity will be provided for discussion and edits. The Delphi round results broken down by stakeholder group as well as the UG’s feedback on the characteristic and specification pairs will be made available to aid discussion. Meeting participants will be asked to vote on the inclusion of each characteristic and specification pair, with a supermajority of >70% among voting participants needed for inclusion in the final TPP. An abstention will be permitted.

#### Recruitment

Participants in the Delphi study will be asked whether they wish to be considered for participation. Meeting attendees will be agreed upon with the SAG and invited to the meeting by email.

#### Sampling

Purposive sampling will be used to ensure there are at least 2 members from all the key stakeholder groups previously identified, with the exception of ML-ARIAS developers, to avoid conflicts of interest. Travel expenses will be offered to all attendees.

## Results

This project has received funding from the NIHR Birmingham Biomedical Research Centre since February 2022 and an NIHR incubator grant for regulatory science awarded to the University Hospitals Birmingham NHS Foundation Trust in June 2023.

Both the SMG and SAG have been assembled and have convened their first meetings. Phase 1 began in April 2023; database searches were performed in May 2023 and the phase was completed in November 2023. Phase 2 began in November 2023 and is expected to be completed in March 2024. As of March 2024, 21 interviews have been performed. Phase 3 is expected to begin in April 2024 and be completed in July 2024. The final TPP and its methods will be submitted to a peer-reviewed journal for publication and reported using the Consolidated Criteria for Reporting Qualitative Research (COREQ) criteria [59].

## Discussion

### Benefits of Establishing a TPP for an ML-ARIAS for Use in E-DES

ML-ARIAS offer an innovative approach for E-DES to meet rising demand and improve clinical, economic, or service outcomes. However, there is a risk that any or all of these could be negatively impacted by the introduction of an ML-ARIAS due to failures to design, evaluate, or deploy such tools appropriately. For example, placing an ML-ARIAS designed to be highly sensitive but poorly specific as a primary grader may necessitate additional decision arbitration in the screening service, or risk increasing unnecessary referrals to hospital eye services. Either eventuality may significantly reduce (or even negate) any cost benefits, or negatively impact the experience of people with diabetes or E-DES professionals. Alternatively, an ML-ARIAS’s economic value could be significantly reduced by any requirement to update existing IT infrastructure, making it economically unviable.

While it is impossible to forecast all such potential pitfalls, some are predictable and could be avoided by outlining the NHS’ requirements with regard to an ML-ARIAS within a TPP. This can then be used to guide product development and evaluation. Depending on who is involved in TPP development, these documents can also reflect the priorities of all stakeholder groups, including those often neglected in traditional procurement processes, such as patients and delivery staff.

The knowledge of what should go into a TPP for an ML-ARIAS likely already exists, albeit spread over members of different stakeholder groups and sometimes not formally recorded. Where desired characteristics are not clearly understood, for example, when trade-offs exist between competing priorities, our proposed consensus methodology provides a means to better establish requirements or areas where future research is needed.

Given that product requirements can be hard to identify, it is unsurprising that innovation frequently aligns poorly with the actual needs of health care professionals, health services, and their users. Creating a TPP to gather this knowledge in a concise format can accelerate the development of products that meet these stakeholders’ needs at a cost much less than that of late-stage product failures or unsuccessful deployments. As well as improving developers’ efficiency in product development or testing, other stakeholders stand to gain from TPPs’ development. Knowing the essential characteristics of a tool, commissioners can make more assured commissioning decisions, drawing on the collective knowledge of all stakeholders contributing to the TPP. In an ML-ARIAS context, people with diabetes will also have a unique opportunity to influence product design and implementation strategies, increasing the likelihood that tools are acceptable to them and good DES uptake is maintained.

### Comparison to Previous Work

To our knowledge, this project represents the first public use of TPPs for an AI health care technology. Our final TPP and the learning accrued through its development will provide a strong basis for the development of TPPs for other disease areas. As no best practice currently exists on TPP development [45], we will use a 3-phase multistakeholder-modified Delphi-consensus method similar to that used to develop reporting guidelines [56,60], core outcome sets [57,61] and previous TPPs [35,40,62]. This method aims to foster values such as inclusivity, patient empowerment, and consensus, and we intend to transfer these values into best practices in a new field.

### Limitations

This project has a number of potential risks that we have sought to mitigate, most notably a failure to recruit and retain participants in the Delphi process, the omission of critical characteristics, and a failure to adequately represent and balance the needs of stakeholder groups.

With regard to securing good recruitment and retention for the Delphi process, we have secured the strong engagement of all relevant stakeholder groups with representatives of each on the SAG. Their involvement in the design of the Delphi survey aims to ensure it is broadly acceptable and achievable by all, as well as mitigate against a significant “drop-off” between Delphi rounds, a common issue that can introduce bias. To further mitigate this, we will also monitor returns collectively and at a stakeholder group level during each round, allowing interventions such as reminders, further engagement activities, or the extension of round durations, if necessary.

The risk of omitting an essential characteristic in our TPP is increased as this will be the first developed for an AI diagnostic test, and characteristics cannot simply be copied from those previously published in the field. Additionally, some characteristics may only be identified and prioritized by single stakeholder groups. It is therefore important that the TPP development process provide sufficient opportunity for these novel or underappreciated characteristics to be identified. To do this, our reviews will specifically target resources in the field of digital and AI health care to establish which characteristics to report, and our method will provide opportunities for members of all stakeholder groups to put forward additional requirements at multiple stages.

There is also the risk that our methods may generate characteristics that put undue emphasis on the priorities of 1 stakeholder group over another. One may hypothesize that a developer might wish to set TPP requirements at a low, easily achievable level that would be unacceptable to patients and health service providers on the grounds of safety or quality. Conversely, patients and health professionals may have an unrealistic idea of what is possible and argue for TPP requirements to be set near or at perfection (eg, “must not miss any cases”). Our TPP development process seeks to both surface and balance competing priorities to produce a common, achievable target where possible, including being open regarding which stakeholder groups hold different views, potential conflicts of interest, and the actual consequences of decisions and compromises. To ensure that the requirements of the TPP remain achievable, ML-ARIAS developers will be invited to participate in the internet-based Delphi rounds as well as the UG, feedback from which will be presented at the meeting where the final TPP will be agreed. Bidirectional thresholds will also be used in the Delphi phase, such that characteristic and specification pairs must have a minimum level of support as well as a maximum level of dissent allowed to progress, increasing the likelihood that these are achievable as well as acceptable to all.

### Conclusions

In developing a TPP for an ML-ARIAS, we will for the first time bring patients, health care professionals, commissioners, methodologists, clinical AI experts, and developers together to provide a target for AI developers to work toward. It is our aim that this will increase the likelihood of the development of ML-ARIAS that are fit-for-purpose for the NHS, improve screening outcomes and of benefit all stakeholders. In addition, we hope that this first public use of TPPs in health care AI and our open sharing of our methods will enable others to develop TPPs for a range of unmet needs, providing clarity to developers and accelerating innovation toward products that will be welcomed by patients and providers alike. 

## Appendix

Multimedia Appendix 1 Review search strategies.

## Abbreviations

AI: artificial intelligence
ARIAS: automated retinal imaging analysis software
COREQ: Consolidated Criteria for Reporting Qualitative Research
CQC: Care Quality Commission
DES: diabetic eye screening
DR: diabetic retinopathy
E-DES: English diabetic eye screening
ESF: Evidence Standards Framework
MHRA: Medicines and Healthcare Products Regulatory Agency
ML: machine learning
ML-ARIAS: machine learning–automated retinal imaging analysis software
NASSS: nonadoption, abandonment, scale-up, spread, and sustainability
NICE: National Institute for Health and Care Excellence
NIHR: National Institute for Health and Care Research
NSC: National Screening Committee
SAG: study advisory group
SMG: study management group
TPP: target product profile
UG: user group

## Data Availability

Data sharing is not applicable to this article, as no data sets were generated or analyzed during this study.
