# Supplementary material for: Target Product Profile for a Machine Learning–Automated Retinal Imaging Analysis Software for Use in English Diabetic Eye Screening: Protocol for a Mixed Methods Study
Source: JMIR Res Protoc. 2024 Mar 27;13:e50568. doi: 10.2196/50568 (PMC11007610; doi:10.2196/50568)
Supplement: Multimedia Appendix 1 [file resprot_v13i1e50568_app1.docx]

## Appendix 1 – Review search strategies

### Review of systematic reviews of diagnostic test TPPs

### Databases

MEDLINE (OVID), EMBASE (OVID), Web of Science (Clarivate), CINAHL (EBSCO), Epistemonikos

### MEDLINE search strategy : other database searches will be translated from this

Ovid MEDLINE(R) ALL

1 review.pt.

2 (medline or medlars or embase or pubmed).tw,sh.

3 scisearch.tw,sh.

4 cinahl.tw,sh.

5 ((hand adj2 search$) or (manual$ adj2 search$)).tw,sh.

6 (electronic database$ or bibliographic database$ or computeri#ed database$ or online database$).tw,sh.

7 (retraction of publication or retracted publication).pt.

8 or/2-7

9 1 and 8

10 (meta-analys$ or meta analys$ or metaanalys$).tw,sh.

11 (systematic$ adj5 review$).tw,sh.

12 (systematic$ adj5 overview$).tw,sh.

13 (quantitativ$ adj5 review$).tw,sh.

14 (quantitativ$ adj5 overview$).tw,sh.

15 (quantitativ$ adj5 synthesis$).tw,sh.

16 (methodologic$ adj5 review$).tw,sh.

17 (methodologic$ adj5 overview$).tw,sh.

18 (integrative research review$ or research integration).tw.

19 or/10-18

20 9 or 19 549952

21 "target product profil*".ti,ab,kw.

22 QTPP.ti,ab,kw.

23 "quality by design".ti,ab,kw.

24 or/21-23

25 20 and 24

### Systematic review of digital health/AI TPPs

### Databases

MEDLINE, EMBASE, Web of Science (Clarivate), ACM Digital library

### MEDLINE search strategy : other database and web search strategies will be adapted from this

Ovid MEDLINE(R) ALL

1 "quality by design".ti,ab,kw.

2 "target product profil*".ti,ab,kw.

3 QTPP*.ab,kw,ti.

4 or/1-3

5 exp Internet/

6 (online or web or internet or digital*).ti,ab,kw.

7 ((online or web or internet or digital*) adj3 (based or application* or intervention* or program* or therap*)).ti,ab,kw.

8 exp Informatics/

9 exp Medical Informatics/

10 exp Nursing informatics/

11 exp Medical Informatics Computing/

12 exp Consumer Health Informatics/

13 exp Public Health Informatics/

14 exp Medical Informatics Applications/

15 informatics.ti,ab,kw.

16 exp Information Technology/

17 exp Electronic Health Records/

18 exp Medical Records Systems, Computerized/

19 Automated Medical Record*.ti,ab,kw.

20 computeri#ed medical record*.ti,ab,kw.

21 EHR*.ti,ab,kw.

22 Electronic Medical Record*.ti,ab,kw.

23 electronic patient record*.ti,ab,kw.

24 personal health record*.ti,ab,kw.

25 electronic health record*.ti,ab,kw.

26 EMR*.ti,ab,kw.

27 personal health record*.ti,ab,kw.

28 exp Health Information Systems/

29 health information system*.ti,ab,kw.

30 exp Information Systems/

31 exp Computers/

32 exp Precision Medicine/

33 precision medicine.ti,ab,kw.

34 exp Software/

35 software.ti,ab,kw.

36 SaMD.ti,ab,kw.

37 exp Big Data/

38 big data.ti,ab,kw.

39 exp Therapy, Computer-Assisted/

40 exp internet-based intervention/ or social media/

41 exp Digital Technology/

42 virtual health*.ti,ab,kw.

43 health technolog*.ti,ab,kw.

44 "health 2.0".ti,ab,kw.

45 (cyber-medicine* or cybermedicine*).ti,ab,kw.

46 (epatient* or e-patient*).ti,ab,kw.

47 digital health*.ti,ab,kw.

48 (personal adj3 digital).ti,ab,kw.

49 digital medicine*.ti,ab,kw.

50 or/5-49

51 exp Mobile Applications/

52 (app or apps).ti,ab,kw.

53 (mobile application* or App-based).ti,ab,kw.

54 51 or 52 or 53

55 exp Artificial Intelligence/

56 ((artificial* or deep*) adj1 (intelligen* or learn* or smart*)).ti,ab,kw.

57 ((computat* or computer* or machine) adj1 (intelligen* or smart*)).ti,ab,kw.

58 (expert system* adj3 (intelligen* or smart*)).ti,ab,kw.

59 ((Artificial* or intelligen*) adj2 (comput* or technol* or network* or imaging or digital*)).ti,ab,kw.

60 Generative adversarial network.ti,ab,kw.

61 (Algorith* adj5 (intelligen* or artificial* or smart* or automat*)).ti,ab,kw.

62 (Intelligen* adj2 (analys* or analyz* or system* or screen* or informatic* or application* or app or apps or process* or simulation* or interactive* or interaction*)).ti,ab,kw.

63 ((decision tree* or random forest* or knowledge representation* or computer vision system* or computer reasoning* or natural language processing* or connectionist model*) adj2 artificial*).ti,ab,kw.

64 (AI not (aromatase inhibitor* or anatomic insertion* or AI-IgG or apnea index or anti-infective or angiogenesis inhibitors)).ti,ab,kw.

65 (machinelearn* or machine-learn*).ti,ab,kw.

66 exp neural networks, computer/

67 (neural network* adj1 (artificial* or convolutional or deep*)).ti,ab,kw.

68 exp Diagnosis, Computer-Assisted/ and (intelligen* or smart or artificial*).ti,ab,kw.

69 exp Diagnosis, Computer-Assisted/ and (accura* adj2 (diagnos* or detect* or assess* or class*)).ti,ab,kw.

70 exp Diagnosis, Computer-Assisted/ and (automat* adj2 (system* or CAD system* or methodolog* or screen* or identif* or local* or class* or detect* or diagnos*)).ti,ab,kw.

71 exp Therapy, Computer-Assisted/ and (intelligen* or smart or artificial* or automat*).ti,ab,kw.

72 ((assist* or aid* or diagnos* or screen*) adj2 (intelligen* or artificial* or smart* or automat*)).ti,ab,kw.

73 55 or 56 or 57 or 58 or 59 or 60 or 61 or 62 or 63 or 64 or 65 or 66 or 67 or 68 or 69 or 70 or 71 or 72

74 exp Telemedicine/

75 (telemedicine* or tele-medicine* or remote medicine*).ti,ab,kw.

76 (tele-health* or telehealth* or remote health*).ti,ab,kw.

77 (telecare* or tele-care* or remote care).ti,ab,kw.

78 exp Remote Consultation/

79 ((remote* or tele*) adj2 (patient monitor* or consult* or deliver* or intervention* or treatment* or diagnos* or service* or program* or prevent*)).ti,ab,kw.

80 exp Videoconferencing/

81 (videoconferenc* or video-conferenc* or videoconsult* or video-consult*).ti,ab,kw.

82 or/74-81

83 exp Cell Phone/

84 (phone* or telephone* or smartphone* or cellphone* or cell-phone* or smartwatch*).ti.

85 ((phone* or telephone* or smartphone* or cellphone* or smartwatch*) adj3 (based or application* or intervention* or program* or therap*)).ab.

86 (Smartphone* or smart-phone*).ti,ab,kw.

87 (Cell-phone* or cellphone*).ti,ab,kw.

88 Mobile-phone*.ti,ab,kw.

89 exp Computers, Handheld/

90 exp Text Messaging/

91 ((text* or txt) adj3 message*).ti,ab,kw.

92 (SMS or MMS).ti,ab,kw.

93 mhealth.ti,ab,kw.

94 mobile comput*.ti,ab,kw.

95 (m-health or mhealth or m-therapy or mtherapy or mobile health*).ti,ab,kw.

96 ((mobile* or internet*) adj2 intervention*).ti,ab,kw.

97 (mobile health or mhealth or m-health or ehealth or e-health or emental or e-mental).ti.

98 ((mobile health or mhealth or m-health or ehealth or e-health or emental or e-mental) adj3 (based or application* or intervention* or program* or therap*)).ab.

99 (mobile* adj3 (based or application* or intervention* or device* or technolog*)).ti,ab,kw.

100 ((mhealth or m-health or ehealth or e-health or technology enabled care or digital health* or digital medicine* or eportal* or e-portal* or digital portal* or virtual health* or mobile health* or mobile intervention* or mobile comput* or Internet of Things or IoT or epatient* or e-patient* or online or web* or internet or zoom) adj3 (service* or program* or intervention* or deliver* or remote* or treatment* or diagnos* or prevent* or therap* or setting* or session* or remote* or train* or technolog*)).ti,ab,kw.

101 mobile health*.ti,ab,kw.

102 mobile intervention*.ti,ab,kw.

103 (mobile adj3 (comput*or device* or application* or technolog* or platform* or software*)).ti,ab,kw.

104 ((App or Apps) not (mutant or mice or amyloid precursor protein* or gene dose* or acute phase protein*)).ti,ab,kw.

105 (tablet* adj3 (application* or device* or technolog* or platform* or mobile* or comput* or software*)).ti,ab,kw.

106 (mobile application* or Appstore or App-based).ti,ab,kw.

107 or/83-106

108 exp Wearable Electronic Devices/

109 wearable*.ti,ab,kw.

110 108 or 109

111 50 or 54 or 73 or 82 or 107 or 110

112 4 and 111
